# Supplementary material for: Maternal Separation Impairs Cocaine-Induced Behavioural Sensitization in Adolescent Mice
Source: PLoS One. 2016 Dec 9;11(12):e0167483. doi: 10.1371/journal.pone.0167483 (PMC5147915; doi:10.1371/journal.pone.0167483)
Supplement: S1 File — Table A- Raw data for body weight of mice exposed to SN and MSEW rearing conditions. Table B—Raw data for cocaine-induced locomotor sensitization of mice exposed to SN and MSEW rearing conditions. Table C—Raw data for cocaine-induced conditioned place preference of mice exposed to SN and MSEW rearing conditions. Table D—Raw data for cocaine-induced operant self-administration of mice exposed to SN and MSEW rearing conditions. Table E—Raw data for dopamine system analysis of mice exposed to SN and MSEW rearing conditions. (DOCX) [file pone.0167483.s001.docx]

**Supporting Information S1**

**Table A. Raw data for body weight of mice exposed to SN and MSEW rearing conditions.**

| **Body weight** | | | **SN** | **MSEW** |
| --- | --- | --- | --- | --- |
| **Body weight (g)** | PD10 | Mean | 7,12 | 5,15 |
|  |  | SD | 1,47 | 0,63 |
|  |  | SEM | 0,44 | 0,19 |
|  | PD17 | Mean | 9,32 | 7,53 |
|  |  | SD | 2,56 | 1,22 |
|  |  | SEM | 0,77 | 0,37 |
|  | PD30 | Mean | 31,13 | 31,85 |
|  |  | SD | 2,54 | 2,54 |
|  |  | SEM | 0,77 | 0,76 |
|  | PD62 | Mean | 41,55 | 42,57 |
|  |  | SD | 3,17 | 6,32 |
|  |  | SEM | 0,96 | 1,91 |
|  | PD83 | Mean | 45,24 | 47,56 |
|  |  | SD | 4,30 | 7,40 |
|  |  | SEM | 1,30 | 2,23 |

**Table B. Raw data for cocaine-induced locomotor sensitization of mice exposed to SN and MSEW rearing conditions.**

| **Cocaine-induced locomotor sensitization** | | | **SN** | | **MSEW** | |
| --- | --- | --- | --- | --- | --- | --- |
|  |  |  | **Saline** | **Cocaine** | **Saline** | **Cocaine** |
| **Locomotor activity** | Breaks/15 min Day 3 | Mean | 3290,17 | 2829,92 | 2732,67 | 3261,92 |
|  |  | SD | 879,7 | 836,7 | 632,67 | 1420,73 |
|  |  | SEM | 253,95 | 241,53 | 182,64 | 410,13 |
|  | Breaks /15 min Day 5 | Mean | 3333,5 | 8851,25 | 3069,25 | 6401,08 |
|  |  | SD | 962,2 | 2897,33 | 1492,62 | 2210,27 |
|  |  | SEM | 277,76 | 836,39 | 430,88 | 638,05 |
|  | Breaks /15 min Day 13 | Mean | 3367 | 5237,08 | 2758,33 | 4389,67 |
|  |  | SD | 1138,05 | 1434,68 | 905,36 | 1556,09 |
|  |  | SEM | 328,53 | 414,16 | 261,36 | 449,2 |
|  | Breaks /15 min Day 14 | Mean | 7033,33 | 11299,83 | 6431,33 | 8836,5 |
|  |  | SD | 2193,14 | 2531,03 | 1737,25 | 2086,82 |
|  |  | SEM | 633,1 | 730,65 | 501,5 | 602,41 |
| **Stereotypes** | Breaks /15 min Day 3 | Mean | 3446,17 | 3994,58 | 3079,92 | 3523,67 |
|  |  | SD | 360,17 | 793,52 | 336,87 | 533,87 |
|  |  | SEM | 103,97 | 229,07 | 97,24 | 154,11 |
|  | Breaks /15 min Day 5 | Mean | 3843,75 | 5015,58 | 3542,92 | 4430,67 |
|  |  | SD | 431,4 | 435,99 | 476,33 | 597,29 |
|  |  | SEM | 124,53 | 125,86 | 137,5 | 172,42 |
|  | Breaks /15 min Day 13 | Mean | 3743,58 | 4233,67 | 3577,5 | 3894,33 |
|  |  | SD | 333,03 | 292,46 | 577,53 | 325,17 |
|  |  | SEM | 96,14 | 84,43 | 166,72 | 93,87 |
|  | Breaks /15 min Day 14 | Mean | 4584,42 | 5558,08 | 4215,08 | 4746,58 |
|  |  | SD | 587,54 | 538,6 | 642,85 | 435,05 |
|  |  | SEM | 169,61 | 155,48 | 185,58 | 125,59 |

**Table C. Raw data for cocaine-induced conditioned place preference of mice exposed to SN and MSEW rearing conditions.**

| **Conditioned place preference paradigm** | | | **SN** | **MSEW** |
| --- | --- | --- | --- | --- |
| **CPP Score** | Saline | Mean | 32,79 | 30,60 |
|  |  | SD | 198,03 | 133,35 |
|  |  | SEM | 48,03 | 30,59 |
|  | Cocaine, 1,5 mg/kg | Mean | 62,18 | 70,67 |
|  |  | SD | 133,54 | 37,06 |
|  |  | SEM | 42,23 | 13,10 |
|  | Cocaine, 3 mg/kg | Mean | 165,42 | 114,65 |
|  |  | SD | 132,98 | 121,19 |
|  |  | SEM | 44,33 | 40,40 |
|  | Cocaine, 15 mg/kg | Mean | 184,12 | 92,60 |
|  |  | SD | 127,54 | 86,42 |
|  |  | SEM | 42,51 | 27,33 |

**Table D. Raw data for cocaine-induced operant self-administration of mice exposed to SN and MSEW rearing conditions.**

| **Operant self-administration procedure** | | | **SN** | **MSEW** |
| --- | --- | --- | --- | --- |
| **Day 1** | Active hole | Mean | 4,8 | 7,11 |
|  |  | SD | 3,967 | 4,54 |
|  |  | SEM | 1,25448 | 1,51 |
|  | Inactive hole | Mean | 2,7 | 4,67 |
|  |  | SD | 2,21 | 1,73 |
|  |  | SEM | 0,70013 | 0,58 |
| **Day 2** | Active hole | Mean | 4,5 | 7,56 |
|  |  | SD | 0,1 | 5,83 |
|  |  | SEM | 0,03 | 1,94 |
|  | Inactive hole | Mean | 3,1 | 2,22 |
|  |  | SD | 2,56 | 1,39 |
|  |  | SEM | 0,81 | 0,46 |
| **Day 3** | Active hole | Mean | 6,9 | 5,89 |
|  |  | SD | 4,7 | 4,68 |
|  |  | SEM | 1,49 | 1,56 |
|  | Inactive hole | Mean | 3 | 1,56 |
|  |  | SD | 2,26 | 2,07 |
|  |  | SEM | 0,71 | 0,69 |
| **Day 4** | Active hole | Mean | 8,3 | 7,78 |
|  |  | SD | 7,62 | 5,54 |
|  |  | SEM | 2,41 | 1,85 |
|  | Inactive hole | Mean | 3,2 | 2 |
|  |  | SD | 2,86 | 2,45 |
|  |  | SEM | 0,9 | 0,82 |
| **Day 5** | Active hole | Mean | 7,7 | 8,33 |
|  |  | SD | 3,83 | 5,34 |
|  |  | SEM | 1,21 | 1,78 |
|  | Inactive hole | Mean | 0,9 | 1,56 |
|  |  | SD | 1,2 | 1,67 |
|  |  | SEM | 0,38 | 0,56 |
| **Day 6** | Active hole | Mean | 8,4 | 9 |
|  |  | SD | 4,65 | 8,53 |
|  |  | SEM | 1,47 | 2,84 |
|  | Inactive hole | Mean | 1,6 | 1,89 |
|  |  | SD | 1,07 | 2,03 |
|  |  | SEM | 0,34 | 0,68 |
| **Day 7** | Active hole | Mean | 7,7 | 9 |
|  |  | SD | 4,35 | 8,46 |
|  |  | SEM | 1,37 | 2,82 |
|  | Inactive hole | Mean | 1,3 | 1,11 |
|  |  | SD | 1,89 | 1,27 |
|  |  | SEM | 0,6 | 0,4 |
| **Day 8** | Active hole | Mean | 7,3 | 9,22 |
|  |  | SD | 3,4 | 7,93 |
|  |  | SEM | 1,08 | 2,64 |
|  | Inactive hole | Mean | 1,8 | 1,11 |
|  |  | SD | 1,75 | 0,78 |
|  |  | SEM | 0,55 | 0,25 |
| **Day 9** | Active hole | Mean | 9,4 | 12,56 |
|  |  | SD | 4,84 | 10,69 |
|  |  | SEM | 1,53 | 3,56 |
|  | Inactive hole | Mean | 1,7 | 1,67 |
|  |  | SD | 1,95 | 1,41 |
|  |  | SEM | 0,62 | 0,47 |
| **Day 10** | Active hole | Mean | 8,6 | 12,22 |
|  |  | SD | 4,93 | 9,8 |
|  |  | SEM | 1,56 | 3,27 |
|  | Inactive hole | Mean | 1,2 | 1,44 |
|  |  | SD | 1,62 | 2,01 |
|  |  | SEM | 0,51 | 0,67 |

**Table E. Raw data for dopamine system analysis of mice exposed to SN and MSEW rearing conditions.**

| **Dopamine system analysis** | | | **SN** | **MSEW** |
| --- | --- | --- | --- | --- |
| **DAT** | Saline | Mean | 99,99900 | 59,38 |
|  |  | SD | 0,00002 | 17,18 |
|  |  | SEM | 0,00001 | 8,59 |
|  | Cocaine, 3 mg/kg | Mean | 80,83 | 142,80 |
|  |  | SD | 46,08 | 68,17 |
|  |  | SEM | 20,61 | 34,08 |
|  | Cocaine, 15 mg/kg | Mean | 94,19 | 212,55 |
|  |  | SD | 39,55 | 95,02 |
|  |  | SEM | 19,78 | 47,51 |
| **D2R** | Saline | Mean | 100,04 | 44,95 |
|  |  | SD | 0,10 | 13,93 |
|  |  | SEM | 0,01 | 6,97 |
|  | Cocaine, 3 mg/kg | Mean | 90,80 | 41,54 |
|  |  | SD | 34,94 | 12,27 |
|  |  | SEM | 4,22 | 6,13 |
|  | Cocaine, 15 mg/kg | Mean | 68,58 | 113,96 |
|  |  | SD | 35,22 | 57,43 |
|  |  | SEM | 6,15 | 25,69 |
| **DA** **Turnover** | Saline | Mean | 4,13 | 5,24 |
|  |  | SD | 1,63 | 2,47 |
|  |  | SEM | 0,67 | 1,10 |
|  | Cocaine, 3 mg/kg | Mean | 2,55 | 3,82 |
|  |  | SD | 1,83 | 1,42 |
|  |  | SEM | 0,82 | 0,64 |
|  | Cocaine, 15 mg/kg | Mean | 2,65 | 9,04 |
|  |  | SD | 0,48 | 5,00 |
|  |  | SEM | 0,24 | 2,23 |
| **Pitx3** | Saline | Mean | 99,99 | 127,80 |
|  |  | SD | 44,45 | 29,84 |
|  |  | SEM | 19,88 | 13,34 |
|  | Cocaine, 3 mg/kg | Mean | 63,15 | 165,67 |
|  |  | SD | 16,17 | 62,90 |
|  |  | SEM | 8,08 | 31,45 |
|  | Cocaine, 15 mg/kg | Mean | 74,76 | 78,63 |
|  |  | SD | 36,90 | 15,93 |
|  |  | SEM | 18,45 | 7,97 |
| **Nurr1** | Saline | Mean | 114,43 | 206,28 |
|  |  | SD | 29,56 | 54,45 |
|  |  | SEM | 14,78 | 24,35 |
|  | Cocaine, 3 mg/kg | Mean | 130,38 | 87,94 |
|  |  | SD | 62,63 | 19,57 |
|  |  | SEM | 31,32 | 9,78 |
|  | Cocaine, 15 mg/kg | Mean | 117,91 | 105,24 |
|  |  | SD | 25,25 | 9,25 |
|  |  | SEM | 11,29 | 4,63 |
