# Supplementary material for: Correction: Maternal Separation Impairs Cocaine-Induced Behavioural Sensitization in Adolescent Mice
Source: PLoS One. 2017 Apr 25;12(4):e0176788. doi: 10.1371/journal.pone.0176788 (PMC5404837; doi:10.1371/journal.pone.0176788)
Supplement: S2 File — (PDF) [file pone.0176788.s002.pdf]

PLOS | ONE

RESEARCH ARTICLE

Maternal Separation Impairs Cocaine-Induced Behavioural Sensitization in Adolescent Mice

Irene Gracia-Rubio[1], Elena Martinez-Laorden[2,3], Maria Moscoso-Castro[1], M. Victoria Milanés[2,3], M. Luisa Laorden[2,3], Olga Valverde[1,4]*

1 Neurobiology of Behavior Research Group (GReNeC). Department of Experimental and Health Sciences, Universitat Pompeu Fabra, Barcelona, Spain, **2** Group of Cellular and Molecular Pharmacology, Faculty of Medicine, University of Murcia, Murcia, Spain, **3** Instituto Murciano de Investigación Biosanitaria (IMIB), Murcia, Spain, **4** Neuroscience Research Program. IMIM (Hospital del Mar Research Institute) Barcelona, Spain

* olga.valverde@upf.edu

CrossMark
click for updates

OPEN ACCESS

Citation: Gracia-Rubio I, Martinez-Laorden E, Moscoso-Castro M, Milanés MV, Laorden ML, Valverde O (2016) Maternal Separation Impairs Cocaine-Induced Behavioural Sensitization in Adolescent Mice. PLoS ONE 11(12): e0167483. doi:10.1371/journal.pone.0167483

Editor: James Edgar McCutcheon, University of Leicester, UNITED KINGDOM

Received: September 26, 2016

Accepted: November 15, 2016

Published: December 9, 2016

Copyright: © 2016 Gracia-Rubio et al. This is an open access article distributed under the terms of the Creative Commons Attribution License, which permits unrestricted use, distribution, and reproduction in any medium, provided the original author and source are credited.

Data Availability Statement: All relevant data are within the paper and raw data from experiments are available as Supporting Information.

Funding: This study was supported by UE MedBioinformatics project (Grant Agreement Number: 634143), MINECO (SAF2013-41761-R-FEDER and SAF2013-49076-P-FEDER), Spanish Ministry of Health (Retic-ISCIII-RD/12/0028/0024-FEDER and RETICS-ISCIII-RD 12/0028/003-FEDER and Plan Nacional sobre Drogas 2014/020), Generalitat de Catalunya (2014SGR34) and Fundación Séneca (15405/PI/10), Región de

Abstract

Adverse early-life conditions induce persistent disturbances that give rise to negative emotional states. Therefore, early life stress confers increased vulnerability to substance use disorders, mainly during adolescence as the brain is still developing. In this study, we investigated the consequences of maternal separation, a model of maternal neglect, on the psychotropic effects of cocaine and the neuroplasticity of the dopaminergic system. Our results show that mice exposed to maternal separation displayed attenuated behavioural sensitization, while no changes were found in the rewarding effects of cocaine in the conditioned place preference paradigm and in the reinforcing effects of cocaine in the self-administration paradigm. The evaluation of neuroplasticity in the striatal dopaminergic pathways revealed that mice exposed to maternal separation exhibited decreased protein expression levels of D2 receptors and increased levels of the transcriptional factor Nurr1. Furthermore, animals exposed to maternal separation and treated with cocaine exhibited increased DA turnover and protein expression levels of DAT and D2R, while decreased Nurr1 and Pitx3 protein expression levels were observed when compared with saline-treated mice. Taken together, our data demonstrate that maternal separation caused an impairment of cocaine-induced behavioural sensitization possibly due to a dysfunction of the dopaminergic system, a dysfunction that has been proposed as a factor of vulnerability for developing substance use disorders.

Introduction

Adverse early life conditions have been associated with brain development alterations [1] increasing vulnerability to psychiatric disorders throughout life such as depression or substance use disorder [2–4]. In this situation, maternal separation with early weaning has been proposed as an early life stress model that produces behavioural alterations related to mood disorders in adolescent mice that persist in adulthood [5,6]. Animal studies support the notion that acute or chronic exposure to stress facilitates the initiation and escalation of drug abuse [7]. Therefore, recent theories propose that drugs of abuse are used in efforts to self-medicate during emotional disorders to relieve feelings of sadness and anhedonia [8]. Indeed, increasing

Murcia. IG-R was funded by FPI fellowship BES-2011-046655 associated to SAF2010-15793.

Competing Interests: The authors have declared that no competing interests exist.

evidence in humans shows that depressive states are likely determinants of drug use and abuse vulnerability [9]. In addition, adolescence is a critical period in which the main brain areas involved in cognitive and emotional skills are still developing [10]. Moreover, the mesocorticolimbic dopamine (DA) system, one of the most critical neural systems in processing salient events, is subject to changes during adolescence [11,12]. In this system, DA neurons project from the ventral tegmental area (VTA) to the nucleus accumbens (NAc), which is an important substrate for rewarding experiences together with other brain areas including the amygdala, the hippocampus and the prefrontal cortex [13,14]. Interestingly, several transcriptional factors regulate the homeostasis of the DA system including the orphan nuclear receptor-related factor 1 (Nurr1) and the paired-like homeobox 3 gene (Pitx3) [15,16]. Nurr1 activates the transcription of the DA transporter (DAT), the vesicular monoamine transporter 2 (VMAT2), and tyrosine hydroxylase (TH), the rate limiting enzyme in the synthesis of DA [15]. Additionally, the expression of Nurr1 is controlled by DA signalling, mainly through D2 DA receptor (D2R) activation. Pitx3 is an essential modulator of Nurr1-mediated transcription in midbrain DA neurons and a key factor for specification of the DA neurons phenotype [15]. Experimental studies also showed that cocaine use induces neuroadaptive changes in cellular and synaptic functions, including alterations in the DA system [17]. Several studies have tried to elucidate the link between emotional disorders and substance use disorder, but few reports have evaluated the effects of chronic stress and drugs of abuse during adolescence in rodents [18,19]. Hence, in this study, we investigated the influence of maternal separation on cocaine-induced behavioural effects, including locomotor sensitization and reward in adolescent mice. We also evaluated cocaine-induced modifications in the dopaminergic system to elucidate the neuroplastic alterations in mice exposed to adverse early-life experiences. We thus used CD1 male mice to evaluate the effects of maternal separation with early weaning and standard nest on cocaine-induced sensitization to locomotor activity, the rewarding effects of cocaine in the conditioned place preference (CPP), and the reinforcing properties of cocaine by means of the self-administration paradigm. Furthermore, protein expression levels of DAT, D2R and DA turnover and the transcriptional factors Nurr1 and Pitx3 were evaluated in the NAc and VTA, respectively, of mice exposed to MSEW and SN rearing conditions under basal conditions and after the exposure to intermittent cocaine treatment.

Materials and methods

Animals

We used 36 male and 36 female outbred CD1 adult mice purchased in Charles River, Barcelona, Spain, as breeders. For breeding, mice were housed in pairs in standard cages in a temperature- (21 ± 1˚C), humidity- (55% ± 10%), and light-cycle-controlled room. The room was lit between 8:00 h and 20:00 h, and experiments were conducted during the light phase (8:30 to 15:00 h) except for the self-administration procedure, in which the room was lit from 20:00 to 8:00 h. Food and water were available *ad libitum* except during behavioural experiments. All procedures were conducted in accordance with European guidelines (BOE-2013-1337; Directive 2010-63EU) regulating animal research, and were approved by the local ethic committee ("Comité Etico de Experimentación Animal"—Universitat Pompeu Fabra and Barcelona Biomedical Research Park) and for Government of Catalonia ("Generalitat de Catalunya").

Rearing conditions

The rearing conditions were conducted as described [6]. Briefly, mice were randomly assigned to two different experimental groups, standard nest (SN) and maternal separation with early weaning (MSEW) groups respectively. In the MSEW group, offspring were separated from

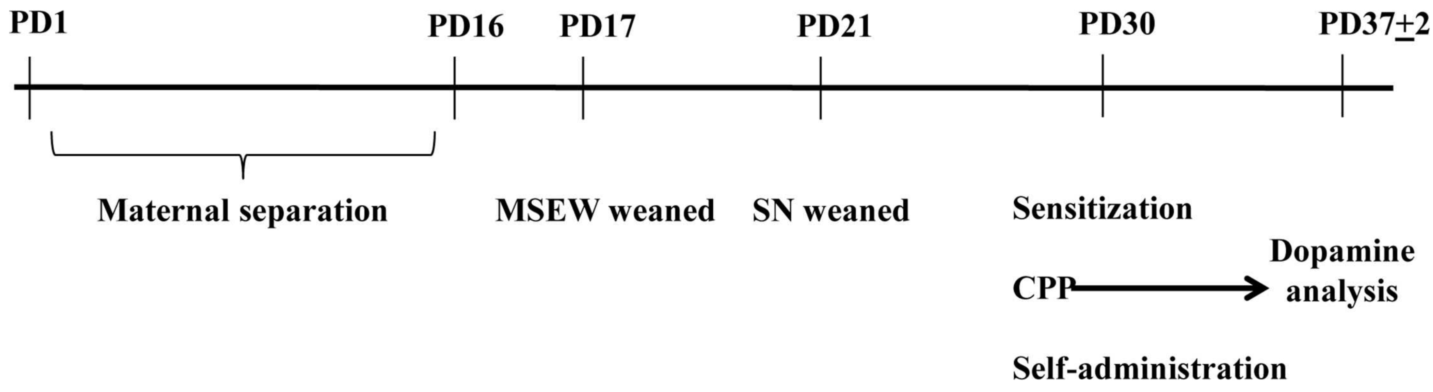

Fig 1. Schematic representation of the experimental procedure.

doi:10.1371/journal.pone.0167483.g001

their mothers for 4 h per day on postnatal day (PD) 2–5 (9:30–13:30 h) and 8 h per day on PD6-16 (9:30–17:30 h) [5,6]. During the maternal separation procedure, dams were separated from the pups, whereas pups remained in the home cage. To ensure an appropriate body temperature, electric blankets were placed under home cages. Pups from the MSEW group were weaned at PD17, while SN-group pups remained with their mothers and were not weaned until PD21. No death of pups was observed during the whole procedure of maternal separation. After weaning, the offspring were housed in groups of 4 animals of the same sex and only male mice were used in the subsequent experiments (Fig 1).

We evaluated the effects of rearing conditions on body weight at different postnatal days. Therefore, body weight was measured in the two experimental groups: SN and MSEW groups of mice at PD10, PD17, PD30, PD62 and PD83. Two-way ANOVA calculated for body weight showed an effect of the day ($F_{(4,80)} = 604.87$; $p < 0.01$), without rearing group effect ($F_{(1,20)} = 0.05$; NS), and without interaction between these two factors ($F_{(4,80)} = 1.511$; NS) (Fig 2) (Table A in S1 File).

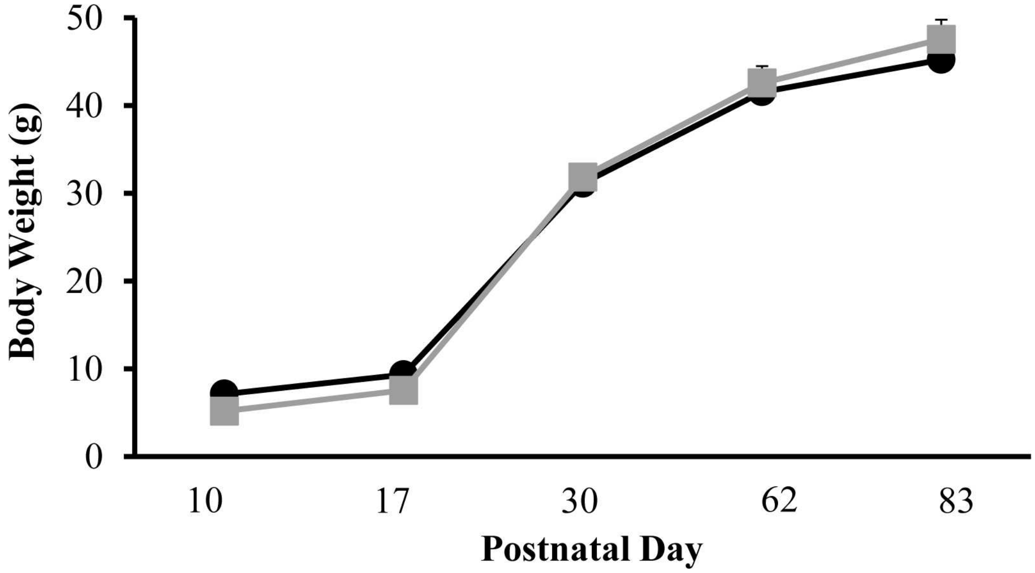

Fig 2. Effects of rearing conditions on body weight at different postnatal days. Body weight was evaluated at at PD10, PD17, PD30, PD62 and PD83 in male mice. Black circles represent SN group of mice and grey squares represent MSEW group of mice. Data are expressed as mean of body weight (g) ± SEM; N = 11–12 mice per group.

doi:10.1371/journal.pone.0167483.g002

Drug and injection procedures

Cocaine was purchased from Alcatel (Ministry of Health, Madrid, Spain) and was dissolved in sterile physiological saline (0.9%). A 7.5 mg/kg dose of cocaine was injected intraperitoneally (i.p.) for behavioural sensitization experiments, 1.5, 3, and 15 mg/kg (i.p.) for the CPP paradigm experiments, and a 1 mg/kg/infusion intravenously (i.v.) for the self-administration study.

Cocaine-induced locomotor sensitization procedure

The sensitization to hyperlocomotor responses elicited by cocaine was evaluated in adolescent mice (PD30±2) on the basis of a previously described procedure [20] with minor modifications. Locomotor activity boxes (24 x 24 x 24 cm) (LE8811 IR, Panlab s.l.u., Barcelona, Spain) were used in a low luminosity room (20 lux) with white noise. On the first day, mice were handled and weighed. The following day, mice were injected with saline and their basal locomotor activity was recorded for 15 min. On each of the following five days, mice were injected with cocaine (7.5mg/kg, i.p.) or saline, and immediately placed in the locomotor activity boxes to record locomotor activity for 15 min. After the repeated cocaine treatment, mice remained in their home cage for five days with no further treatment. After this period, on day 13, the mice were administered a saline injection and the conditioned response associated to the locomotor boxes context was evaluated. On day 14, the mice received a cocaine challenge (7.5 mg/kg, i.p.) and the behavioural sensitization to cocaine was subsequently evaluated. Steretypical behaviour was also measured and analysed during sensitization procedure.

Conditioned place preference paradigm

The rewarding effects of cocaine (1.5, 3 and 15 mg/kg, i.p.) were assessed in adolescent male mice (PD30±2) using the CPP paradigm, as previously described [21]. The CPP was carried out in a box consisting of two square chambers (20 cm x 20 cm x 20 cm) connected by a triangular grey compartment (20 cm x 20 cm x 20 cm). The two compartments differed in colour and floor texture. This procedure was conducted in three different phases, as follows:

Pre-conditioning Phase. The first phase involved a 20 min. free exploration of the boxes in which each mouse was allowed to explore both compartments. Mice showing preference or aversion for one of the compartments (more than 70% or minus than 30%) were excluded from the experiment

Conditioning Phase. For the following six days, mice were injected with cocaine or saline on alternate days. Control animals were administered saline every day. In all cases, after the injection, each mouse was immediately placed into the assigned compartment for 30 min.

Testing Phase. On day 8, mice were allowed to explore both compartments for 20 min, similarly to the pre-conditioning phase. The time spent in each compartment was registered. A score was calculated as the difference of time spent in the compartment associated to the drug in the testing phase and the pre-conditioning.

Operant self-administration procedure

Surgery for the intravenous catheter implantation. 30 adolescent mice (PD30±2) of each rearing group were anesthetized with ketamine/xylazine (100 mg/kg, 20 mg/kg, respectively) and subsequently implanted with an indwelling i.v. silastic catheter as previously described [22,23]. Briefly, a 4.5 cm-long silastic tube (0.3 mm inner diameter, 0.64 mm outer diameter, SilasticR, Dow Corning, Houdeng-Goegnies, Belgium) was adapted to a 22-gauge steel cannula (Semat; Herts; England) curved at a 90° angle and then placed in a cement disk

(Dentalon plus, Heraeus Kulzer, Germany). The catheter tubing was inserted 1.4 cm into the right jugular vein (limited with a dab of silicone) and held with suture. The rest of the catheter passed subcutaneously (s.c.) from the insertion position to the cannula that was fixed in the back of mice, and the incision was closed with staples. All incisions were coated with Betadine (Meda Pharma SAU, S. Fernando de Henares, Madrid, Spain). Mice were treated with analgesics (meloxicam; 0.5mg/kg; s.c.; Inflacam, Chanelle Ltd, Loughrea, Co., Galway, IE) and antibiotic (Enrofloxacino; 7.5 mg/kg; i.p.; Batryl, Bayer Hispania S.L, Sant Joan Despí, Barcelona, Spain) in order to maximize their recovery. Following surgery, mice were allowed to recover for 3–5 days prior to beginning the self-administration procedure.

Apparatus for self-administration experiments. The self-administration experiments were carried out in mouse operant chambers (Model ENV-307A-CT, Medical Associates, Georgia, VT, USA) containing two holes; one was defined as active and the other as inactive. Nose-poking into the active hole produced a cocaine infusion (reinforcement) that was paired with two stimulus lights, one of which was placed inside the nose-poke and the other above the active hole. Nose-poking into the inactive hole had no consequences. The side on which the active/inactive hole was placed was counterbalanced. The chambers were housed in sound- and light-attenuated boxes provided with fans to provide ventilation and white noise.

Cocaine self-administration procedure. Cocaine self-administration sessions were performed as previously described [22,23] with minor modifications. Responding was maintained by cocaine (1 mg/kg/infusion) delivered in 20μl over 2s. Cocaine was infused using a syringe set on a microinfusion pump (PHM-100A, Med-Associates, Georgia, VT, USA) and connected via Tygon tubing (0.96 mm outer diameter, Portex Fine Bore Polythene Tubing, Portex Limited, Kent, England) to a liquid swivel (375/25, Instech Laboratories, Plymouth Meeting, PA, USA) and to the catheters implanted in the mice. Self-administration sessions (1 h daily) were conducted for 10 consecutive days. To begin with, the box light was on for 3 s and turned off for the rest of the session. The session began with a priming infusion of the drug. Mice were trained under a fixed ratio 1 (FR1) schedule of reinforcement. The number of reinforces was limited to 50 per session to avoid overdose and each reinforcement was followed by a 30 s time-out period where nose-poking into the active hole had no consequences. A mouse was considered to have learned the task when the number of responses in the active hole was at least 5, exceeded 75% of the inactive hole response and maintained a stable response with less than a 30% deviation from the mean of the total number of cocaine infusions obtained in two consecutive days (70% of stability).

Dopamine system analysis

Preparation of tissue extract. Adolescent mice (PD37±2) were sacrificed by decapitation 1 h after the CPP test. Brains were rapidly removed and stored immediately at -80˚C prior to western blotting (DAT, DR2, Nurr1 and Pitx3) and high performance liquid chromatography (HPLC) analysis (DA and 3,4-dihydroxyphenylacetic acid (DOPAC) levels). Brains were sliced on a cryostat and kept at -20˚C until each region of interest came into the cutting plane. NAc and VTA were micro-punched from frozen brain sections (500 μm), sectioned using a cryostat, in accordance with the mice brain atlas [24]. All micro-punched samples were frozen and stored at -80˚C.

Electrophoresis and Western Blotting. Western blotting analyses were performed as described previously [25,26], therefore, bilateral punches from NAc and VTA were placed in a buffer containing phosphate buffered saline, 10% sodium dodecyl sulfate (SDS), protease inhibitors (Boehringer Mannheim, Mannheim, Germany) and a phosphatase inhibitor Cocktail Set (Calbiochem, Darmstadt, Germany), homogenized and sonicated for 30 s prior to

centrifugation at 6.000g for 10 min at 4°C. Samples containing 20 μg of protein were loaded on a 10% SDS/polyacrylamide gel, electrophoresed and transferred onto polyvinylidene difluoride membranes (Millipore, Bedford, MA, USA). Nonspecific binding of antibodies was prevented by incubating the membranes in 1% bovine serum albumin (BSA) in Tris-buffered saline Tween-20 (TBST; 10 mM Tris HCl, pH 7.6, 150 mM NaCl, 0.15% Tween 20). The blots were incubated at 4°C overnight with the following primary antibodies: rabbit polyclonal anti-Nurr1 (1:500; sc-991, Santa Cruz Biotechnology, Santa Cruz, CA, USA); rabbit polyclonal anti-Pitx3 (1:750; ab30734, Abcam, Cambridge, UK); rat monoclonal anti-DAT (1:2000; MAB369, Millipore), and rabbit polyclonal anti D2R (1:500;AB5084P, Millipore). Goat anti-rabbit immunoglobulin G (IgG), Horseradish peroxidase (HRP)-linked (1:5000; sc-2004, Santa Cruz, Biotechnology) or goat anti-rat IgG, HRP-linked (1:5000;sc-2032, Santa Cruz Biotechnology) were used as secondary antibodies. After washing, immunoreactivity was detected with an enhanced chemiluminescent/chemifluorescent Western blots detection system (ECL Plus, GE Healthcare, Little Chalfont, Buckinghamshire, UK) and visualized by a Typhoon 9410 variable mode Imager (GE Healthcare). Blots were incubated with stripping buffer (glycine 25mM, SDS 1%, pH2) for 1 hour at 37°C and subsequently reblocked and probed with rabbit polyclonal antiglyceraldehyde 3-phosphate dehydrogenase (GADPH, Cell Signalling Technology Inc., Danvers, MA, USA) or α-tubulin (Cell Signalling Technology Inc.), which were used as loading controls. The ratios DAT/GADPH, D2R/GADPH, Nurr1/α-tubulin and Pitx3/α-tubulin were plotted and analysed. Proteins levels were corrected for individual levels.

Estimation of DA and its metabolite DOPAC. DA and DOPAC were determined in the NAc by HPLC with electrochemical detection as previously described [27]. One punch from each animal was obtained and added to 60 μl of a solution containing 1M $HClO_4$ (Sigma Chemical Co) and 2.7 mM ethylenediaminete-triacetic acid (Sigma Chemical Co). The samples were homogenized by slight sonication for approximately 1 min, centrifuged (6000xg; 4°C) for 10 min and the supernatants were taken for analysis and filtered through ultra-free MC 0.22 μm filter (Millipore). The pellets were re-suspended by adding 100 μl of 1N NaOH. The total amount of proteins from each sample was subsequently measured by spectrophotometry. From each sample 10 μl were injected into a 5-μm C18 reversed-phase column (Waters, Milford, MA, USA) through a Rheodyne syringe-loading injector 200 μL loop (Waters). The mobile phase consisted of a 95% (v/v) mixture of water and methanol with sodium acetate (50 mM; Sigma Chemical Co), citric acid (20 mM; Sigma Chemical Co), L-octyl-sodium sulfonate (3.75 mM; Sigma Chemical Co), di-n-butylamine (1 mM; Sigma Chemical Co) and EDTA (0.135 mM; Sigma Chemical Co), adjusted to pH 4.3. Chromatographic data were analysed with Millenium 2010 Chromatography Manager Equipment (Millipore). DA and DOPAC were simultaneously detected and quantified by reference to calibration curves run at the beginning of the assays. The content of DA and DOPAC is expressed as ng/mg protein. The DA turnover was determined as the DOPAC/DA ratio. The DOPAC/DA ratio was used as an index of DA metabolism.

Statistical Analysis

Data from behavioural sensitization and self-administration experiments were analysed using a three-way ANOVA with repeated measures. Factors of variation were day, rearing groups and treatment for the sensitization study, and day, rearing groups and nose-pokes for the self-administration study. Data from body weight were evaluated using a two-way ANOVA with rearing groups and days as factors of variation or one-way ANOVA (rearing conditions) in the case of self-administration experiments. Data from CPP, western blotting and DA turnover analysis were evaluated using a two-way ANOVA with rearing groups and treatment as factors of variation. Correlations between Nurr1/Pitx3 expressions, DAT and DRD2 protein levels,

were assessed using Pearson's rank correlation. Bonferroni post-hoc tests were applied as required. Results are given as mean ± SEM, and differences were considered significant when the probability of error was less than 5%. All the analyses were performed using the SPSS (version 19).

Results

Cocaine-induced behaviour sensitization

Locomotor sensitization induced by cocaine (7.5 mg/kg) was evaluated in adolescent male mice (PD30±2). No differences were found in the basal locomotor activity between rearing groups (data not shown). As shown in Table 1, three-way ANOVA indicated an effect of the rearing group, cocaine treatment and day effects with interaction between rearing group and day, treatment and day, and between all three factors. The Bonferroni post-hoc test showed that acute (day 3) and repeated cocaine administration (day 7) increased locomotor activity in SN and MSEW groups of mice compared with the saline control group ($p<0.01$). Moreover, the hyperlocomotion induced by repeated doses of cocaine on day 7 was lower in the MSEW group compared with the SN group ($p<0.01$). Hence, only mice from the SN group showed an increase in locomotor responses after repeated treatment with cocaine (effects on day 3 versus day 7) ($p<0.01$). No differences were observed between acute and repeated cocaine effects (effects on day 3 versus day 7) in the MSEW group (Fig 3) (Table B in S1 File). On day 13, mice were evaluated for conditioned hyperlocomotion as a result of repeated exposure to the locomotor activity boxes (contextual hyperlocomotion). All the mice were therefore administered a single injection of saline and locomotor activity was assayed. Contextual hyperlocomotion activity was found to be higher in mice previously treated with cocaine ($p<0.01$), independently of the rearing conditions (Fig 3) (Table B in S1 File). On day 14, all mice received a cocaine challenge (7.5 mg/kg) and were then evaluated in the locomotor activity chambers. Locomotor sensitization was observed in mice exposed to SN rearing conditions when compared with acute (day 3) and repeated cocaine treatment effects (day 7) ($p<0.01$, respectively) (Fig 3) (Table B in S1 File). MSEW mice also increased locomotion after the cocaine challenge (day 14) when compared with the effects observed on day 3 and 7, respectively ($p<0.01$). Nevertheless, behavioural sensitization was attenuated in mice exposed to MSEW rearing conditions versus SN mice ($p<0.01$) (Fig 3) (Table B in S1 File), thus showing that maternal separation impaired the development of neuroadaptations required for behavioural sensitization.

Cocaine-induced stereotypical behaviour was also evaluated during locomotor sensitization procedure in adolescent male mice (PD30±2). Three-way ANOVA indicated an effect of the

Table 1. Three-way ANOVA calculated to evaluate the effect of maternal separation on cocaine-induced locomotor sensitization.

Cocaine-induced locomotor sensitization		
	F	P <
R	$F_{(1,44)} = 4.722$	0.05
T	$F_{(1,44)} = 57.53$	0.01
D	$F_{(3,132)} = 116.8$	0.01
R X T	$F_{(1,44)} = 0.872$	NS
R X D	$F_{(3,132)} = 3.354$	0.05
T X D	$F_{(3,132)} = 8.521$	0.01
R X T X D	$F_{(3,132)} = 4.340$	0.01

Rearing group (R), Treatment (T), Day (D).

doi:10.1371/journal.pone.0167483.t001

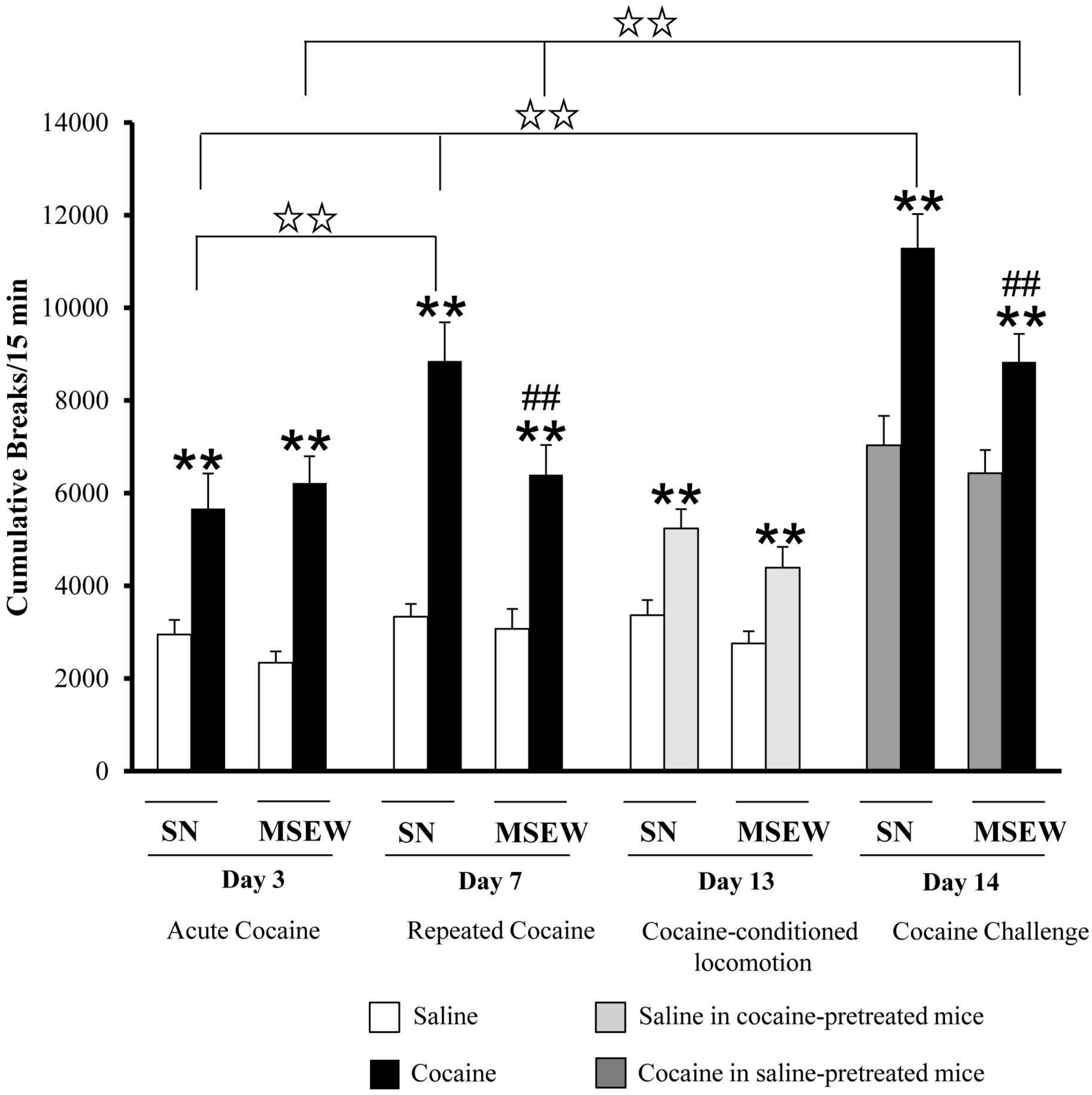

Fig 3. Effects of maternal separation on cocaine-induced locomotor sensitization. Data are expressed as the mean (± SEM) of cumulative breaks/15 min in locomotor activity boxes. N = 12 mice per group. ** p<0.01 saline vs. cocaine on the same days. ## p<0.01 comparisons between rearing groups (SN vs. MSEW). Two white stars p<0.01, day effects as indicated by the lines (Bonferroni post-hoc test).

doi:10.1371/journal.pone.0167483.g003

rearing group ($F_{(1,44)} = 14.638$; $p < 0.01$), the cocaine treatment ($F_{(1,44)} = 5389.7$; $p < 0.01$) and a day effect ($F_{(3,132)} = 104.05$; $p < 0.01$), with interaction between day and treatment ($F_{(3,132)} = 7.09$; $p < 0.01$), and without interaction between all three factors ($F_{(3,132)} = 00486$; NS) (Fig 4) (Table B in S1 File).

Body weights of animals were measured throughout the experiments. A two-way ANOVA was calculated to analyse the body weight of control groups of mice previously exposed to SN or MSEW conditions, and revealed an effect of day.

Body weight of control mice exposed to SN and MSEW rearing conditions was analysed throughout the procedure of behaviour sensitization from PD30 to PD42. Two-way ANOVA

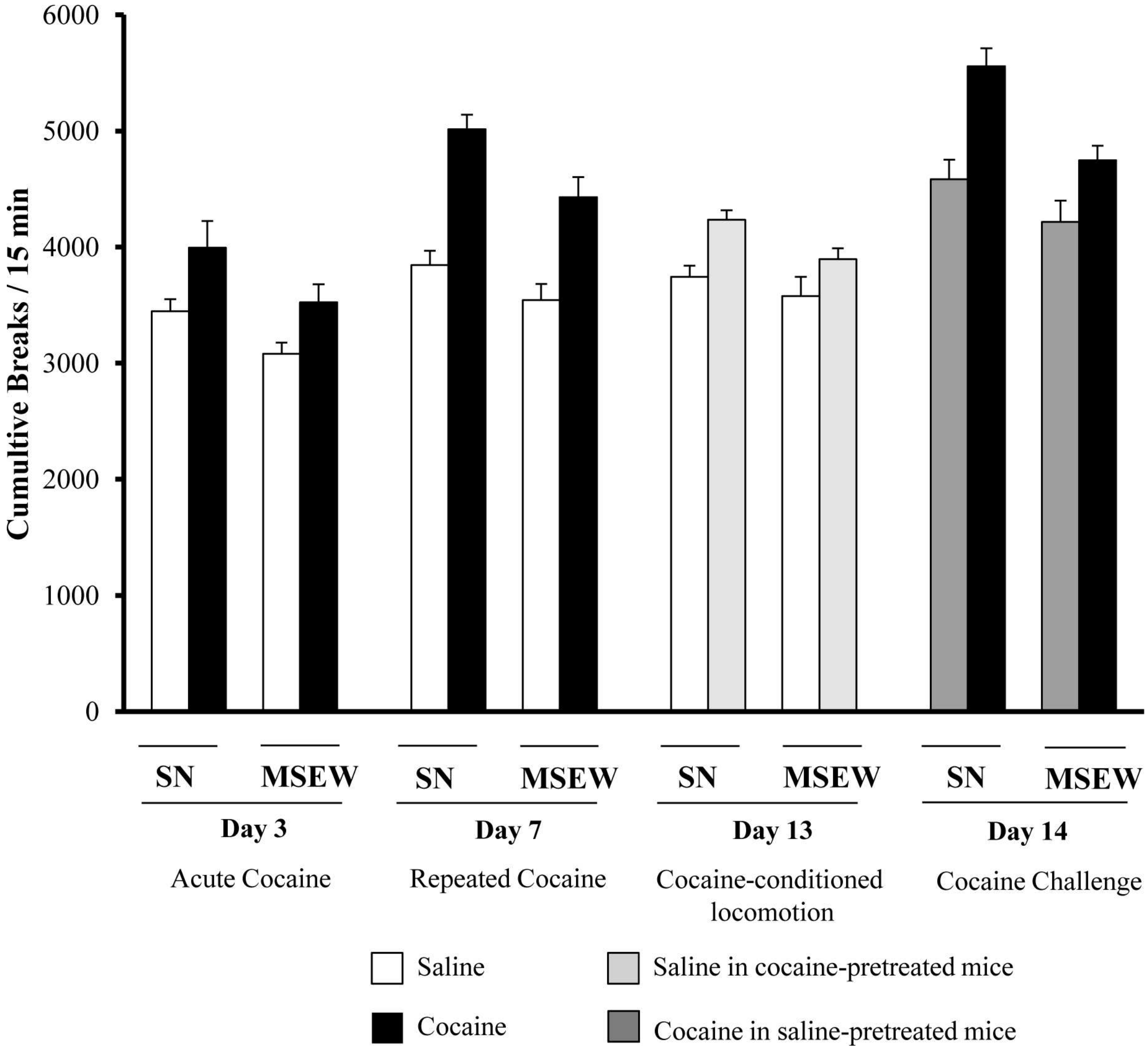

Fig 4. Effects of maternal separation on cocaine-induced stereotypical behaviour during locomotor sensitization procedure. Data are expressed as the mean (± SEM) of cumulative breaks/15 min in locomotor activity boxes. N = 12 mice per group.

doi:10.1371/journal.pone.0167483.g004

revealed an effect of the day ($F_{(7,154)} = 236.18$; $p < 0.01$), without rearing group effect ($F_{(1,22)} = 1.495$; NS) and without interaction between these two factors ($F_{(7,154)} = 1.41$; NS) (data not shown).

Cocaine-induced rewarding effects in the conditioned place preference paradigm

The effect of maternal separation on the rewarding properties of cocaine (1.5, 3 and 15 mg/kg, i.p.) was evaluated in adolescent male mice (PD30±2). Two-way ANOVA showed a treatment effect ($F_{(3,83)} = 6.30$; $p<0.01$), with no rearing group effect ($F_{(1,83)} = 0.34$; NS) or interaction between the two factors ($F_{(3,83)} = 1.538$; NS) (Fig 5) (Table C in S1 File).

Body weight of control mice exposed to SN and MSEW rearing conditions was analysed throughout the CPP procedure from PD30 to PD37. Two-way ANOVA revealed an effect of

the day ($F_{(7,259)} = 272.6$; $p < 0.01$), without rearing group effect ($F_{(1,37)} = 0.263$; NS) and without interaction between these two factors ($F_{(7,259)} = 0.263$; NS) (data not shown).

Cocaine-induced reinforcing effects in the self-administration paradigm

Fig 6 shows the effects of maternal separation on the reinforcing effects of cocaine in the self-administration procedure in adolescent mice (PD30±2). SN and MSEW mice were trained to self-administer cocaine at doses of 1 mg/kg/infusion for 10 days. In accordance with previous established criteria (see Material and methods for details), the percentage of mice achieving the acquisition criteria was 33% for the SN group and 31% for the MSEW group. Three-way ANOVA showed a nose-poke effect and interaction between nose-poke and day (Table 2). Bonferroni post-hoc analysis revealed that the SN group discriminated between active and inactive nose-pokes from day 3 to day 10 (days 3 and 4, $p<0.05$; days 5 to 10, $p<0.01$), whereas the MSEW group discriminated from day 2 to day 10 (days 2 to 10, $p<0.01$). Our results showed no significant differences between rearing groups in the cocaine self-administration study (Fig 6) (Table D in S1 File).

Body weight of control mice exposed to SN and MSEW rearing conditions was analysed at the beginning of the self-administration procedure PD35. The mean of the body weight was 32,1 ± 0,4 g for SN group and 30,4 ± 0,6 g for MSEW group. One-way ANOVA revealed an effect of the rearing conditions ($F_{(1,47)} = 8.350$; $p < 0.01$).

Influence of maternal separation and cocaine administration on levels of DAT and D2R proteins and DA turnover in the NAc

The effects of maternal separation on DAT, D2R and DA turnover were evaluated in mice after performing cocaine-induced CPP (PD37±2). Two-way ANOVA analysis for DAT

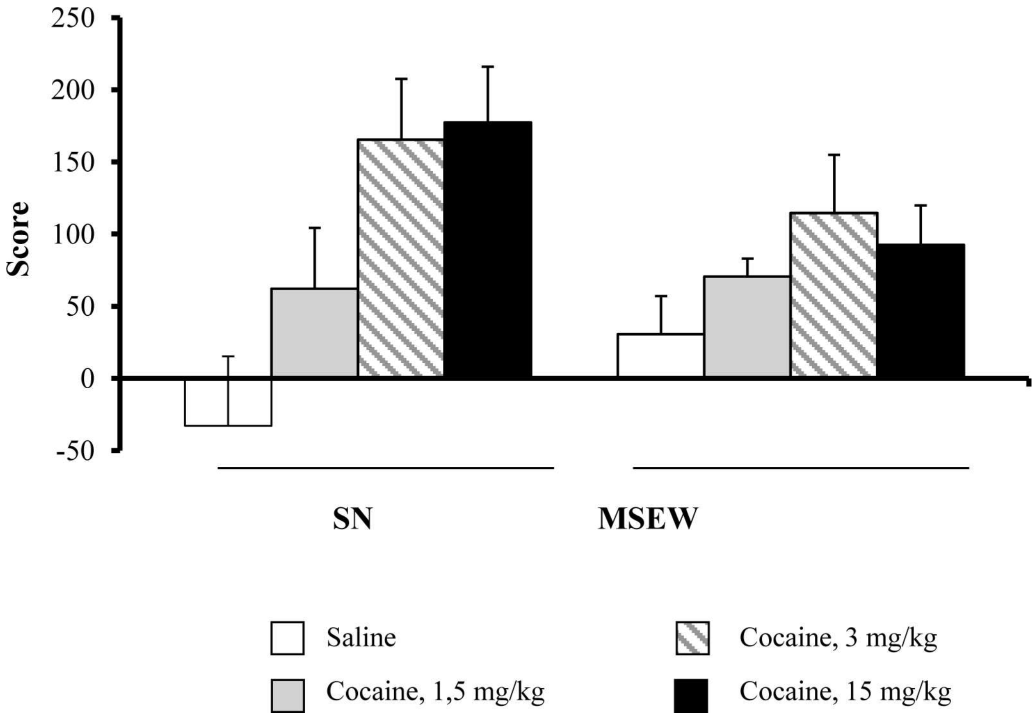

Fig 5. Effects of maternal separation on the rewarding effects of cocaine in the conditioned place preference paradigm. Data are expressed as the mean (± SEM) of the score calculated in the CPP (the difference between the time spent in the compartment associated to the drug in the testing phase versus the pre-conditioning phase). N = 8–15 mice per group.

doi:10.1371/journal.pone.0167483.g005

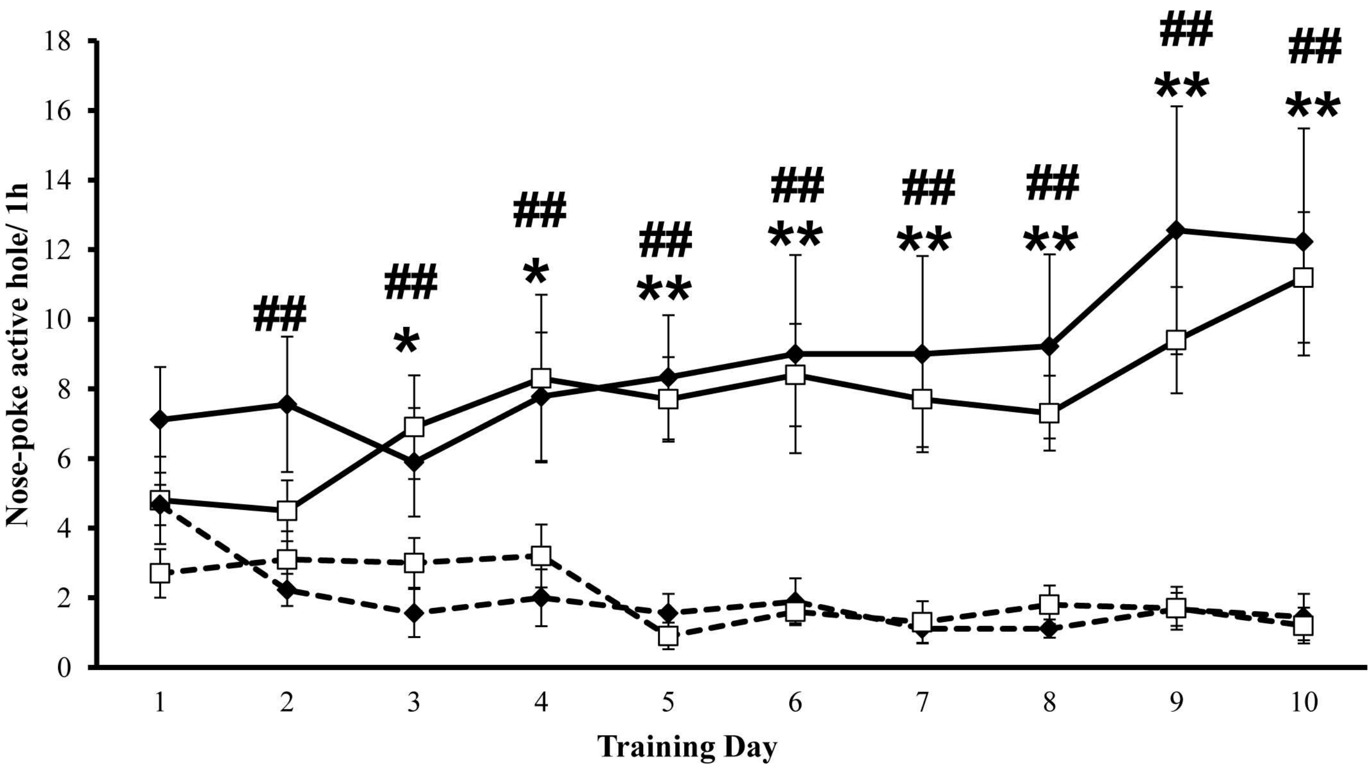

Fig 6. Effects of maternal separation on the reinforcing effects of cocaine in the self-administration procedure. Data are expressed as the mean (± SEM) of nose-pokes into the active/inactive hole throughout the 10 days of training sessions (1 h per session). N = 9–10 mice per group. White symbols with continuous lines represent nose-pokes into the active hole in the SN group and white symbols with dotted lines represent nose-pokes into the inactive hole in the SN group. Black symbols with continuous lines represent nose-pokes into the active hole in the MSEW group, and black symbols with dotted lines represent nose-pokes into the inactive hole. * p<0.05; ** p<0.01 nose-pokes into the active hole vs. inactive hole in the SN group. ## p<0.01 nose-pokes into the active hole vs. inactive hole in the MSEW group (Bonferroni post-hoc test).

doi:10.1371/journal.pone.0167483.g006

protein levels showed a significant effect of the rearing group ($F_{(1,25)} = 5.061$; $p<0.05$), treatment ($F_{(2,25)} = 4.157$; $p<0.05$), and interaction between these factors ($F_{(2,25)} = 5.026$; $p<0.05$). Bonferroni post-hoc analysis revealed no significant effect for the SN group treated with cocaine. However, cocaine significantly increased the levels of DAT in MSEW mice when compared with the saline-treated group ($p<0.05$ and $p<0.01$, with doses of 3 and 15 mg/kg, respectively) (Fig 7A) (Table E in S1 File).

Table 2. Three-way ANOVA calculated to evaluate the effect of maternal separation on cocaine-self-administration.

	Cocaine self-administration	
	**F**	**P <**
**R**	$F_{(1,17)} = 4.722$	NS
**N**	$F_{(1,17)} = 57.53$	0.01
**D**	$F_{(9,153)} = 116.8$	NS
**R X N**	$F_{(1,17)} = 0.872$	NS
**R X D**	$F_{(9,153)} = 3.354$	NS
**N X D**	$F_{(9,153)} = 8.521$	0.01
**R X N X D**	$F_{(9,153)} = 4.340$	NS

Rearing group (R), Nose-poke (N), Day (D).

doi:10.1371/journal.pone.0167483.t002

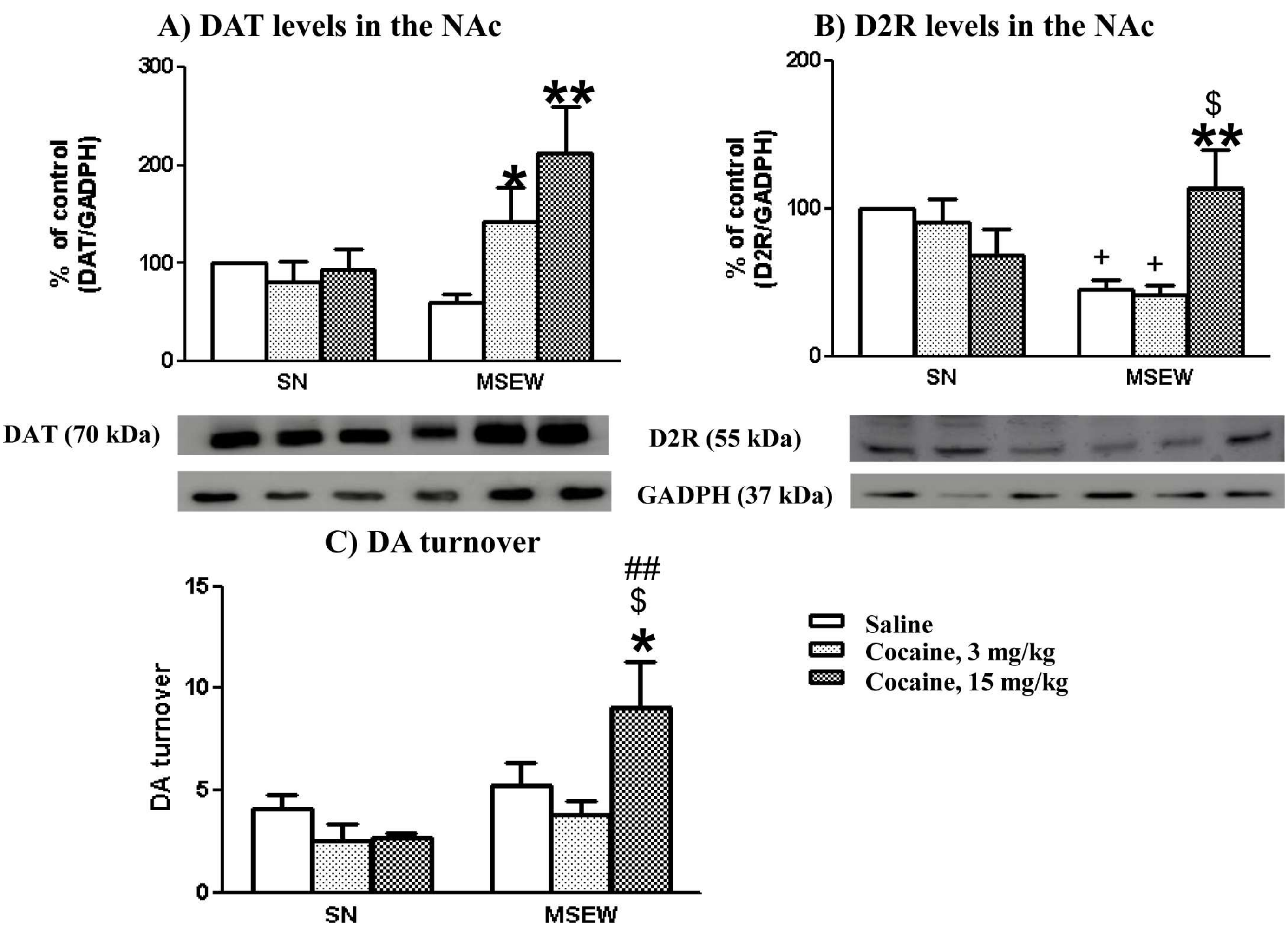

Fig 7. Effect of maternal separation on DAT, D2R levels and DA turnover in the NAc. (**A, B**) Densitometric analysis of specific integrated optical density (percentage of control) signals normalized to the corresponding GADPH levels and representative Western-blotting analysis of DAT and D2R in the NAc. (**C**) DA turnover (as determined by the DOPAC/DA ratio) in the NAc. Each bar corresponds to mean ± SEM, N = 4–6 mice per group. * p<0.05, ** p<0.01 saline vs. cocaine in MSEW group; + p<0.05 vs. SN group treated with saline; $ p<0.05 vs. SN group treated with cocaine (15 mg/kg) (Bonferroni post-hoc test).

doi:10.1371/journal.pone.0167483.g007

Two-way ANOVA for D2R protein levels showed no rearing group effect ($F_{(1,26)} = 2.37$; NS) or treatment effect ($F_{(2,26)} = 1.393$; NS), but did show an interaction between rearing groups and treatment factors ($F_{(2,25)} = 6.502$; $p<0.05$). Bonferroni post-hoc analysis showed that the D2R protein increased in the MSEW group treated with cocaine (15 mg/kg) compared with their saline control groups ($p<0.05$ and $p<0.01$, respectively). The MSEW group treated with saline or cocaine (3 mg/kg) showed a decrease in D2R protein levels ($p<0.05$) when compared with the respective SN group (Fig 7B) (Table E in S1 File).

The state of midbrain dopaminergic neurons was evaluated in the SN and MSEW groups in basal conditions and after cocaine administration during the CPP assay. Thus, DA content (data not shown), DOPAC production (data not shown) and DA turnover (as estimated by the ratio DOPAC/DA) were analysed in the NAc. Two-way ANOVA for DA turnover showed a rearing group effect ($F_{(1,24)} = 9.53$; $p<0.01$), with no treatment effect ($F_{(2,24)} = 2.65$; NS) or interaction between the two factors ($F_{(2,24)} = 3.36$; NS). Bonferroni post-hoc test showed a significant increase in DA turnover in the MSEW group treated with the highest dose of cocaine

when compared with animals treated with saline ($p<0.05$). In addition, DA turnover increased in the MSEW group treated with cocaine (15 mg/kg) when compared with the SN group treated with the same dose of cocaine ($p<0.01$) (Fig 7C) (Table E in S1 File).

Influence of maternal separation and an intermittent treatment of cocaine on the levels of Nurr1 and Pitx3 proteins on VTA

The expression levels of transcriptional factors Nurr1 and Pitx3 in the VTA of mice exposed to cocaine during CPP procedure were investigated (PD37±2). Two-ANOVA for Nurr1 levels showed no rearing group effect ($F_{(1,20)} = 0.64$; NS), but did show a treatment effect ($F_{(2,20)} = 4.74$; $p<0.05$) and interaction between both factors ($F_{(2,20)} = 7.09$; $p<0.01$). The Bonferroni post-hoc test revealed a significant decrease in Nurr1 levels in the MSEW group after cocaine treatment (3 and 15 mg/kg; $p<0.05$ and $p<0.01$, respectively) when compared with the saline control group. Unexpectedly, the comparison between Nurr1 levels in SN versus MSEW saline control groups revealed a significant increase in the level of this protein in the MSEW saline control group ($p<0.05$) (Fig 8A) (Table E in S1 File).

Two-way ANOVA for Pitx3 levels revealed a rearing effect ($F_{(1,20)} = 8.90$; $p<0.05$), no treatment effect ($F_{(2,20)} = 2.76$; NS) and interaction between these two factors ($F_{(2,20)} = 3.92$; $p<0.05$). Bonferroni post-hoc analysis showed a significant decrease in the MSEW group treated with cocaine (15 mg/kg) compared with the MSEW saline control group ($p<0.05$). Comparisons between the MSEW versus SN groups showed a significant increase of Pitx3 levels ($p<0.05$) between rearing groups treated with the lowest doses of cocaine (3 mg/kg) (Fig 8B) (Table E in S1 File).

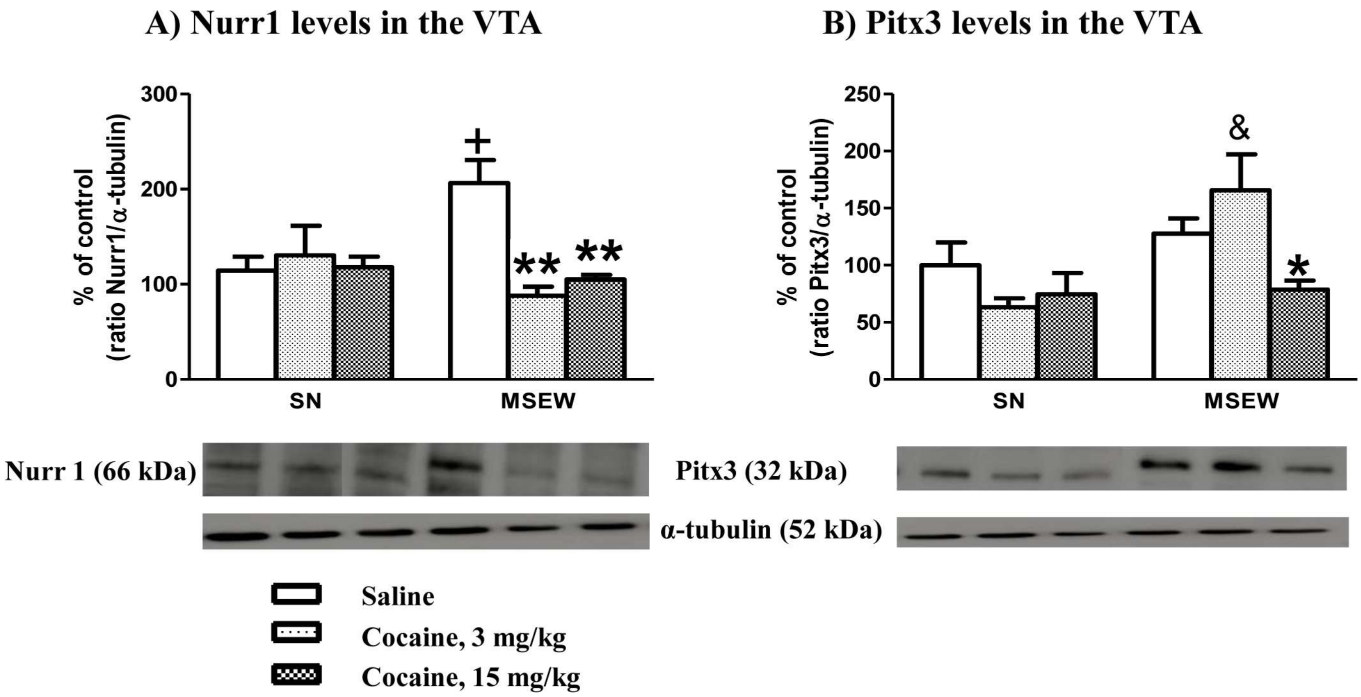

Fig 8. Effects of maternal separation on Nurr1 and Pitx3 levels in the VTA. Densitometric analysis of specific integrated optical density (percentage of control) signals normalized to the corresponding α-tubulin levels and representative Western-blotting analysis of Nurr1 (**A**) and Pitx3 (**B**) levels in the VTA. Each bar corresponds to mean ± SEM, N = 4–5 mice per group. * $p<0.05$, ** $p<0.01$ saline vs. cocaine in the same rearing group (MSEW); + $p<0.05$ SN vs. MSEW; & $p<0.05$ SN vs. MSEW treated with cocaine (3 mg/kg) (Bonferroni post-hoc test).

doi:10.1371/journal.pone.0167483.g008

Relationship between Nurr1 and/or Pitx3 and DAT, and DRD2

Pearson's correlations were calculated to analyse the relationship between the expression of Nurr1 and Pitx3 with DAT and D2R protein levels respectively. No significant correlation was observed between Nurr1 expression and DAT protein levels in a general analysis, although group-based analyses showed that the DAT expression negatively correlated with Nurr1 in MSEW mice treated with saline ($r = -0.938$, $p<0.05$). Moreover, a significant correlation was also found between Nurr1 expression and D2R protein levels ($r = - 0.397$, $p<0.05$). Follow-up group-based analyses revealed that the link between Nurr1 and D2R was found in MSEW mice treated with saline ($r = -0.883$, $p<0.05$). In addition, a slight link between Nurr1 and D2R was observed in SN mice treated with cocaine (15 mg/kg) ($r = -0.825$, $p = 0.085$). No correlation was found between Pitx3 expression and DAT or D2R protein levels (data not shown).

Discussion

Our study shows, for the first time, that adolescent mice exposed to MSEW exhibited a reduction in cocaine-induced psychotropic effects and, particularly, cocaine-induced behavioural sensitization. Our results also demonstrated particular alterations in the dopaminergic neurotransmission in midbrain areas controlling reward. Behavioural and neurochemical alterations are unlike to be due to nutritional changes because no differences in body weight were observed in mice during adolescence accordingly with previous studies [6]. Only mice exposed to self-administration showed significant differences regarding rearing conditions at PD35, probably related to a different capability of the rearing groups to recover from surgery.

The acute administration of cocaine (7.5 mg/kg) induced a similar enhancement of locomotor activity in mice exposed to SN or MSEW rearing conditions. However, the administration of repeated doses of cocaine produced an attenuated behavioural sensitization. Additionally, maternal separation did not affect the cocaine-induced conditioned place preference in adolescent mice.

Previous studies evaluating the influence of maternal separation on such psychostimulant effects showed contradictory results. Thus, whereas some authors reported an increase in cocaine sensitization following maternal separation in adult mice [28,29], other studies did not find differences regarding such a phenomenon in adolescent rats [30]. The attenuated behavioural sensitization here reported may be attributed to a reduced activity in brain reward circuits, in accordance with changes in dopaminergic neurotransmission observed in limbic circuits. A possible consequence of this is a lower sensitivity to the psychotropic effects of cocaine, which may be conducive to repeating the pleasurable experiences induced by the substance, thus increasing the risk of developing a drug use disorder [14,30]. The attenuated cocaine effects in MSEW group of animals could also be influenced by stereotypical behaviour. Indeed, cocaine-induced stereotypical behaviour mainly in SN rearing group (main effect of the ANOVA), but not interaction was found between rearing conditions and cocaine treatment (Fig 4).

In contrast, no differences in cocaine-induced rewarding effects were observed in mice exposed to SN or MSEW rearing conditions in the CPP. A main effect of the ANOVA for treatment indicated a cocaine effect in both rearing groups, but only a trend to an overall more modest effect was shown in MSEW group of mice (Fig 5). Previous studies revealed contradictory results regarding the influence of chronic stress on reward. Therefore, a decrease in amphetamine or morphine rewarding effects was reported in rodents exposed to chronic mild stress [31,32], and a neonatal model of stress produced a decrease in the rewarding effects of cocaine evaluated in the CPP [33]. However, enhanced rewarding effects of cocaine were also found in adolescent mice exposed to stress by social defeat [19]. The discrepancy in these

previous results may be due to the characteristics of stressful situations in terms of the frequency and duration of stress as well as the age at which the stress situation took place. Moreover, most of the previous studies were performed in adult mice, whereas in this study we used mice in early adolescence (PD30±2), which exhibit a greater variability in their behaviours. In fact, some behavioural studies have suggested that adolescents are hyposensitivity to psychostimulants effects relative to adult rats [34,35]. Accordingly, in a recent study evaluating the rewarding effects of cocaine in the conditioned place preference, an effective dose of 25 mg/kg of cocaine revealed a significant effect in adolescent OF1 mice [19]. Conversely, the higher dose used in the present study was 15 mg/kg and only a marginally effect was observed in the CPP. Additionally, we have recently reported, that MSEW mice exhibit anhedonia as assessed in the saccharin test [6]. Consequently, MSEW conditions seem to induce modifications in the rewarding DA system, which alter the psychotropic effects of cocaine.

Preclinical studies have also found that early life stress induced by maternal separation may impair the function of mesolimbic circuits involved in reward; either increasing [29,36] or reducing reinforcing the effects of stimuli, probably due to the development of anhedonia [37,38]. Our data did not show any differences in the reinforcing effects of cocaine in the self-administration paradigm regarding rearing conditions. In this sense, previous studies evaluating the influence of repeated stress have also shown controversial data. Hence, whereas adult social defeat stress resulted in enhanced cocaine self-administration in socially stressed adult mice [39], no differences were found in socially stressed adolescent mice [19]. Therefore, the lack of robust results here reported regarding the rewarding and reinforcing effects of cocaine in the CPP and self-administration paradigm respectively may be also attributed to the fact that we used adolescent mice, showing an important behavioural variability, probably due to brain immaturity [35].

The molecular evaluation of the DA system in adolescent mice exposed to MSEW showed significant changes in several components of this neurotransmitter system, including a decrease in D2R protein expression levels in the NAc and an increase of the transcriptional factor Nurr1 in the VTA. No differences were observed in DAT, DA turnover or the transcriptional factor Pitx3. The decrease in D2R in saline-treated MSEW mice may contribute to supporting the attenuation of cocaine-induced reward here reported. Accordingly, mice exposed to a model of posttraumatic stress disorder showed anhedonia and decreased cocaine intake in the self-administration procedure associated to a D2R decrease in NAc [40]. Furthermore, mice exposed to repeated stressful situations also showed a D2R density reduction in the NAc [41]. It is noteworthy that high levels of D2R have been proposed to confer resilience to developed substance use disorders, whereas low levels of D2R seem to increase vulnerability to such disorders [42]. Hence, the administration of the D2R antagonist, sulpiride (100 mg/kg), reduced cocaine sensitization [43] and blocked cocaine, morphine and alcohol rewarding effects in the CPP [44–46]. Interestingly, knockout mice lacking D2R receptors exhibited a higher cocaine self-administration than wild-type littermates only with high doses of cocaine (3.2 mg/kg, i.v.), whereas no differences were found between genotypes with a lower dosage (0.03 mg/kg, i.v.) [47]. Such data support the absence of cocaine rewarding effects in adolescent MSEW mice, which would probably require higher doses of cocaine to experience reward. Other studies have also evaluated the effects of maternal separation on the dopaminergic function, reporting no differences in D2R density in adult mice [48], while an increased D2R expression was reported using a prenatal stress model in rats [49]. Our results do not identify the particular involvement of pre- and post-synaptic D2Rs, but we hypothesized that both types of receptors could probably participate in the observed effects. MSEW mice treated with saline or a low dose of cocaine (3 mg/kg) showed reduced levels of D2R and DA turnover, whereas MSEW mice treated with higher doses of cocaine (15 mg/kg) displayed increased

D2R expression levels and DA turnover when compared with saline-treated mice. Such data may support the involvement of pre-synaptic D2R regulating the release of DA [50,51]. The modulation of D2R expression in response to cocaine suggests the involvement of post-synaptic D2R [50]. Thus, our biochemical findings seem to be consistent with the behavioural experiments here reported and allow us to provide an explanatory hypothesis for behavioural data. Nevertheless, the complex expression pattern of D2R acting at the pre- and post-synaptic cleft in different neuronal types in the mesolimbic circuit poses difficulties when explaining our results [26]. Our data also show the existence of a compensatory mechanism to enhance the impaired DA activity after maternal separation conditions by increasing the expression of Nurr1 protein levels in the VTA. Indeed, Nurr1 is a transcription factor that is essential for the differentiation of midbrain DA neurons [52], and has been reported to be enhanced in the VTA and substance nigra of mice lacking D2R as a compensatory mechanism [53]. Our results also show a negative correlation between Nurr1 expression levels in the VTA, and D2R and DAT expression levels in the NAc of MSEW mice, supporting the role of Nurr1 as a compensatory mechanism when the DA system is downregulated. Thus, prenatally stressed rats showed increased Nurr1 expression and decreased TH levels in the VTA [49]. Similarly, Nurr1 expression increased in the midbrain of rats with decreased DA contents due to stress exposure [54], which would also support this compensatory Nurr1 mechanism to counteract the reduction of DA levels [55].

SN mice showed no changes in DA turnover or in the expression of proteins DAT, D2R, Nurr1, or Pitx3 after cocaine administration. In our study, brain samples were dissected 48 h after the final cocaine injection when no expected residual psychostimulant effects remained. In such conditions, the decreased expression of Nurr1 and Pitx3 may be viewed as a homeostatic response to increase DA neurotransmission after the cocaine treatment. Furthermore, the lack of modulation of other genes affecting DA neurotransmission after repeated cocaine administration suggests that the partial down-regulation of Nurr1 and Pitx3 is not sufficient to modify the expression of some putative downstream target genes [17].

Our results prompt us to suggest that mice exposed to MSEW developed an impaired function of the DA system as revealed in saline-treated mice. This DA dysfunction is hypothesized to contribute to drug-seeking and drug-taking behaviours [14,56]. Therefore, the intermittent administration of cocaine in MSEW mice was able to elicit neuroadaptive changes in the DA system, which would trigger counteracting mechanisms. Our results propose the existence of increased DAT expression levels in response to an elevated DA release induced by cocaine treatment in MSEW mice. Accordingly, MSEW mice treated with cocaine displayed higher DA turnover and increased protein expression levels of D2R in addition to a decrease in Nurr1 and Pitx3 to compensate the DA neurotransmitter dysfunction.

In conclusion, our results show an attenuation of cocaine-induced sensitization associated to a dopaminergic dysfunction in brain areas controlling reward mechanisms. Such a dysfunction has been proposed as a factor of vulnerability for developing substance use disorders.

Supporting Information

S1 File. Table A- Raw data for body weight of mice exposed to SN and MSEW rearing conditions.

Table B—Raw data for cocaine-induced locomotor sensitization of mice exposed to SN and MSEW rearing conditions.

Table C—Raw data for cocaine-induced conditioned place preference of mice exposed to SN and MSEW rearing conditions.

Table D—Raw data for cocaine-induced operant self-administration of mice exposed to SN

and MSEW rearing conditions.

Table E—Raw data for dopamine system analysis of mice exposed to SN and MSEW rearing conditions.

(DOCX)

Author Contributions

Conceptualization: IG-R EM-L MVM MLL OV.

Formal analysis: IG-R EM-L MM-C MVM MLL OV.

Funding acquisition: MVM MLL OV.

Investigation: IG-R EM-L MM-C.

Methodology: IG-R EM-L MM-C MVM MLL OV.

Project administration: MVM MLL OV.

Resources: MVM MLL OV.

Supervision: MVM MLL OV.

Validation: IG-R EML MM-C MVM MLL OV.

Visualization: IG-R EM-L MM-C MVM MLL OV.

Writing – original draft: IG-R EM-L MM-C MVM MLL OV.

Writing – review & editing: IG-R EM-L MM-C MVM MLL OV.

References

1. Kaufman J, Plotsky PM, Nemeroff CB, Charney DS: Effects of early adverse experiences on brain structure and function: clinical implications. Biol. Psychiatry 2000, 48: 778–790. PMID: 11063974
2. Widom CS, DuMont K, Czaja SJ: A prospective investigation of major depressive disorder and comorbidity in abused and neglected children grown up. Arch. Gen. Psychiat 2007, 64: 49–56. doi: 10.1001/archpsyc.64.1.49 PMID: 17199054
3. Gerra G, Leonardi C, Cortese E, Zaimovic A, Dell'agnello G, Manfredini M, et al: Childhood neglect and parental care perception in cocaine addicts: relation with psychiatric symptoms and biological correlates. Neurosci. Biobehav. Rev 2009, 33: 601–10. doi: 10.1016/j.neubiorev.2007.08.002 PMID: 17904221
4. Lupien SJ, McEwen BS, Gunnar MR, Heim C: Effects of stress throughout the lifespan on the brain, behaviour and cognition. Nat. Rev. Neurosci 2009, 10. 434–445. doi: 10.1038/nrn2639 PMID: 19401723
5. George ED, Bordner KA, Elwafi HM, Simen AA: Maternal separation with early weaning: a novel mouse model of early life neglect. BMC Neurosci 2010, 11: 123. doi: 10.1186/1471-2202-11-123 PMID: 20920223
6. Gracia-Rubio I, Moscoso-Castro M, Pozo OJ, Marcos J, Nadal R, Valverde O: Maternal separation induces neuroinflammation and long-lasting emotional alterations in mice. Prog. Neuropsychopharmacol. Biol. Psychiatry 2016, 65: 104–17. doi: 10.1016/j.pnpbp.2015.09.003 PMID: 26382758
7. Sinha R: Chronic stress, drug use, and vulnerability to addiction. Ann. N. Y. Acad. Sci 2008, 1141: 105–30. doi: 10.1196/annals.1441.030 PMID: 18991954
8. Weiss RD, Griffin ML, Mirin SM: Drug Abuse as Self-Medication for Depression: An Empirical Study. Am. J. Drug Alcohol Abuse 1992, 18: 121–9. PMID: 1562010
9. Cheetham A, Allen NB, Yücel M, Lubman DI: The role of affective dysregulation in drug addiction. Clin. Psychol. Re 2010,. 30: 621–34.
10. Crews F, He J, Hodge C: Adolescent cortical development: a critical period of vulnerability for addiction. Pharmacol. Biochem. Behav.2007, 86: 189–99. doi: 10.1016/j.pbb.2006.12.001 PMID: 17222895

11. Everitt BJ, Wolf ME: Psychomotor stimulant addiction: a neural systems perspective. J. Neurosci 2002, 22: 3312–20. PMID: 11978805

12. Burke AR, Miczek KA: Stress in adolescence and drugs of abuse in rodent models: role of dopamine, CRF, and HPA axis. Psychopharmacology (Berl) 2014, 231: 1557–80.

13. Koob GF, Le Moal M: Review. Neurobiological mechanisms for opponent motivational processes in addiction. Philos. Trans. R. Soc. Lond. B. Biol. Sci 2008, 363: 3113–23. doi: 10.1098/rstb.2008.0094 PMID: 18653439

14. Koob GF, Volkow ND: Neurocircuitry of addiction. Neuropsychopharmacology 2010, 35: 217–238. doi: 10.1038/npp.2009.110 PMID: 19710631

15. Reddy SDN, Rayala SK, Ohshiro K, Pakala SB, Kobori N, Dash P: et al: Multiple coregulatory control of tyrosine hydroxylase gene transcription. Proc. Natl. Acad. Sci. USA 2011, 108: 4200–5. doi: 10.1073/pnas.1101193108 PMID: 21368136

16. Bissonette GB, Roesch MR: Development and function of the midbrain dopamine system: What we know and what we need to. Genes, Brain Behav 2015, 15: 62–73.

17. Leo D, di Porzio U, Racagni G, Riva MA, Fumagalli F, Perrone-Capano C: Chronic cocaine administration modulates the expression of transcription factors involved in midbrain dopaminergic neuron function. Exp. Neurol 2007, 203: 472–80. doi: 10.1016/j.expneurol.2006.08.024 PMID: 17070804

18. Doremus-Fitzwater TL, Spear LP: Age-related differences in amphetamine sensitization: effects of prior drug or stress history on stimulant sensitization in juvenile and adult rats. Pharmacol. Biochem. Behav 2010, 96: 198–205. doi: 10.1016/j.pbb.2010.05.005 PMID: 20471409

19. Rodríguez-Arias M., Montagud-Romero S, Rubio-Araiz A, Aguilar MA, Martín-García E., Cabrera R., et al: Effects of repeated social defeat on adolescent mice on cocaine-induced CPP and self-administration in adulthood: integrity of the blood-brain barrier. Addict. Biol 2015, sep 16.

20. Blanco E, Bilbao A, Luque Rojas MJ, Palomino A, Bermúdez-Silva FJ, Suárez J: Attenuation of cocaine-induced conditioned locomotion is associated with altered expression of hippocampal glutamate receptors in mice lacking LPA1 receptors. Psychopharmacology (Berl) 2011, 220: 27–42.

21. Martin M, Ledent C, Parmentier M, Maldonado R, Valverde O: Cocaine, but not morphine, induces conditioned place preference and sensitization to locomotor responses in CB1 knockout mice. Eur. J. Neurosci 2000, 12: 4038–4046. PMID: 11069600

22. Soria G, Mendizábal V, Touriño C, Robledo P, Ledent C, Parmentier M, et al: Lack of CB1 cannabinoid receptor impairs cocaine self-administration. Neuropsychopharmacology 2005, 30: 1670–80. doi: 10.1038/sj.npp.1300707 PMID: 15742004

23. Tourino C, Valjent E, Ruiz-Medina J, Herve D, Ledent C, Valverde O: The orphan receptor GPR3 modulates the early phases of cocaine reinforcement. Br. J. Pharmacol 2012, 167: 892–904. doi: 10.1111/j.1476-5381.2012.02043.x PMID: 22612385

24. Paxinos G, Franklin KBJ: The mouse brain in stereotaxic coordinates, Academic Press 2004.

25. García-Pérez D, López-Bellido R, Rodríguez RE, Laorden ML, Núñez C, Milanés MV: Dysregulation of dopaminergic regulatory mechanisms in the mesolimbic pathway induced by morphine and morphine withdrawal. Brain Struct. Funct 2015, 220: 1901–19. doi: 10.1007/s00429-014-0761-5 PMID: 24706046

26. García-Pérez D, Núñez C, Laorden ML, Milanés MV: Regulation of dopaminergic markers expression in response to acute and chronic morphine and to morphine withdrawal. Addict. Biol 2016, 21: 374–86. doi: 10.1111/adb.12209 PMID: 25522207

27. García-Pérez D, López-Bellido R, Hidalgo JM, Rodríguez RE, Laorden ML, Núñez C, et al: Morphine regulates Argonaute 2 and TH expression and activity but not miR-133b in midbrain dopaminergic neurons. Addict. Biol 2015, 20: 104–19. doi: 10.1111/adb.12083 PMID: 23927484

28. Kikusui T, Faccidomo S, Miczek KA: Repeated maternal separation: Differences in cocaine-induced behavioral sensitization in adult male and female mice. Psychopharmacology (Berl) 2005, 178: 202–210.

29. Matthews K, Robbins TW, Everitt BJ, Caine SB: Repeated neonatal maternal separation alters intravenous cocaine self-administration in adult rats. Psychopharmacology (Berl) 1999, 141: 123–34.

30. Planeta CS, Marin MT: Effect of cocaine on periadolescent rats with or without early maternal separation. Braz. J. Med. Biol. Res 2002, 35: 1367–71. PMID: 12426637

31. Papp M, Willner P, Muscat R: An animal model of anhedonia: attenuation of sucrose consumption and place preference conditioning by chronic unpredictable mild stress. Psychopharmacology (Berl) 1991, 104: 255–9.

32. Valverde O, Smadja C, Roques BP, Maldonado R: The attenuation of morphine-conditioned place preference following chronic mild stress is reversed by a CCKB receptor antagonist. Psychopharmacology (Berl) 1997, 131: 79–85.

33. Hays SL, McPherson RJ, Juul SE, Wallace G, Schindler AG, Chavkin C, et al: Long-term effects of neonatal stress on adult conditioned place preference (CPP) and hippocampal neurogenesis. Behav. Brain Res 2012, 227: 7–11. doi: 10.1016/j.bbr.2011.10.033 PMID: 22061798

34. Adriani W, Laviola G: Elevated levels of impulsivity and reduced place conditioning with d-amphetamine: two behavioral features of adolescence in mice. Behav Neurosci 2003, 117: 695–703. PMID: 12931955

35. Spear LP, Brick J: Cocaine-induced behavior in the developing rat. Behav Neural Biol 1979, 26: 401–15. PMID: 574000

36. Kosten TA, Zhang XY, Kehoe P: Chronic neonatal isolation stress enhances cocaine-induced increases in ventral striatal dopamine levels in rat pups. Brain Res. Dev. Brain Res 2003, 141: 109–16. PMID: 12644254

37. Martini M, Valverde O: A single episode of maternal deprivation impairs the motivation for cocaine in adolescent mice. Psychopharmacology (Berl) 2011, 219: 149–158.

38. O'Connor RM, Moloney RD, Glennon J, Vlachou S, Cryan JF: Enhancing glutamatergic transmission during adolescence reverses early-life stress-induced deficits in the rewarding effects of cocaine in rats. Neuropharmacology 2015, 99: 168–176. doi: 10.1016/j.neuropharm.2015.07.012 PMID: 26187394

39. Han X, Albrechet-Souza L, Doyle MR, Shimamoto A, DeBold JF, Miczek KA Social stress and escalated drug self-administration in mice II. Cocaine and dopamine in the nucleus accumbens. Psychopharmacology (Berl) 2015, 232: 1003–10.

40. Enman NM, Arthur., Ward SJ, Perrine SA, Unterwald EM: Anhedonia, Reduced Cocaine Reward, and Dopamine Dysfunction in a Rat Model of Posttraumatic Stress Disorder. Biol. Psychiatry 2015, 78: 871–879. doi: 10.1016/j.biopsych.2015.04.024 PMID: 26115790

41. Puglisi-Allegra S, Kempf E, Schleef C, Cabib S: Repeated stressful experiences differently affect brain dopamine receptor subtypes. Life Sci 1991, 48: 1263–8. PMID: 2002754

42. Volkow ND, Wang G.-J., Fowler J.S., Thanos P.P.K., Logan J., Gatley S.J., et al: Brain DA D2 receptors predict reinforcing effects of stimulants in humans: replication study. Synapse 2002, 46: 79–82. doi: 10.1002/syn.10137 PMID: 12211085

43. Mattingly BA, Hart TC, Lim K, Perkins C: Selective antagonism of dopamine D1 and D2 receptors does not block the development of behavioral sensitization to cocaine. Psychopharmacology (Berl) 1994, 114: 239–42.

44. Matsuzawa S, Suzuki T, Misawa M, Nagase H: Involvement of dopamine D(1) and D(2) receptors in the ethanol-associated place preference in rats exposed to conditioned fear stress. Brain Res 1999, 835: 298–305. PMID: 10415386

45. Nazarian A, Russo SJ, Festa ED, Kraish M, Quinones-Jenab V: The role of D1 and D2 receptors in the cocaine conditioned place preference of male and female rats. Brain Res. Bull 2004, 63: 295–9. doi: 10.1016/j.brainresbull.2004.03.004 PMID: 15196654

46. Rezayof A, Zarrindast M-R, Sahraei H, Haeri-Rohani AH-R: Involvement of dopamine D2 receptors of the central amygdala on the acquisition and expression of morphine-induced place preference in rat. Pharmacol. Biochem. Behav 2002, 74: 187–97. PMID: 12376167

47. Caine SB, Negus SS, Mello NK, Patel S, Bristow L, Kulagowski J, et al: Role of dopamine D2-like receptors in cocaine self-administration: studies with D2 receptor mutant mice and novel D2 receptor antagonists. J. Neurosci 2002, 22: 2977–88. PMID: 11923462

48. Vazquez V, Weiss S, Giros B, Martres M-P, Daugé V: Maternal deprivation and handling modify the effect of the dopamine D3 receptor agonist, BP 897 on morphine-conditioned place preference in rats. Psychopharmacology (Berl) 2007, 193: 475–86.

49. Katunar MR, Saez T, Brusco A, Antonelli MC: Immunocytochemical expression of dopamine-related transcription factors Pitx3 and Nurr1 in prenatally stressed adult rats. J. Neurosci. Re 2009,. 87: 1014–22.

50. Anzalone A, Lizardi-Ortiz JE, Ramos M, De Mei C, Hopf FW, Iaccarino C, et al: Dual control of dopamine synthesis and release by presynaptic and postsynaptic dopamine D2 receptors. J. Neurosci 2012, 32: 9023–34. doi: 10.1523/JNEUROSCI.0918-12.2012 PMID: 22745501

51. Benoit-Marand M, Borrelli E, Gonon F: Inhibition of dopamine release via presynaptic D2 receptors: time course and functional characteristics in vivo. J. Neurosci 2001, 21: 9134–41. PMID: 11717346

52. Jankovic J, Chen S, Le WD: The role of Nurr1 in the development of dopaminergic neurons and Parkinson's disease. Prog. Neurobiol 2005, 77: 128–38. doi: 10.1016/j.pneurobio.2005.09.001 PMID: 16243425

53. Tseng KY, Roubert C, Do L, Rubinstein M, Kelly MA, Grandy DK, et al: Selective increase of Nurr1 mRNA expression in mesencephalic dopaminergic neurons of D2 dopamine receptor-deficient mice. Brain Res. Mol. Brain Res 2000, 80: 1–6. PMID: 11039723

54. Rojas P, Joodmardi E, Perlmann T, Ogren SO: Rapid increase of Nurr1 mRNA expression in limbic and cortical brain structures related to coping with depression-like behavior in mice. J. Neurosci. Res 2010, 88: 2284–93. doi: 10.1002/jnr.22377 PMID: 20175204

55. Campos-Melo D, Galleguillos D, Sánchez N, Gysling K, Andrés ME: Nur transcription factors in stress and addiction. Front. Mol. Neurosci 2013, 6: 44. doi: 10.3389/fnmol.2013.00044 PMID: 24348325

56. Morgan D, Grant KA, Gage HD, Mach RH, Kaplan JR, Prioleau O, et al: Social dominance in monkeys: dopamine D2 receptors and cocaine self-administration. Nat. Neurosci 2002, 5: 169–74. doi: 10.1038/nn798 PMID: 11802171
